# Jaguar Densities across Human-Dominated Landscapes in Colombia: The Contribution of Unprotected Areas to Long Term Conservation

**DOI:** 10.1371/journal.pone.0153973

**Published:** 2016-05-04

**Authors:** Valeria Boron, Joseph Tzanopoulos, Jenny Gallo, Jorge Barragan, Laura Jaimes-Rodriguez, George Schaller, Esteban Payán

**Affiliations:** 1 Durrell Institute of Conservation and Ecology, University of Kent, Canterbury, United Kingdom; 2 Panthera Colombia, Bogotá, Colombia; 3 La Aurora Civil Society Nature Reserve, Hato Corozal, Colombia; 4 Escuela de Biologia, Universidad Industrial de Santander, Bucaramanga, Colombia; 5 Panthera, New York, New York, United States of America; 6 Wildlife Conservation Society, Bronx, New York, United States of America; Oregon State University, UNITED STATES

## Abstract

Large carnivores such as jaguars (*Panthera onca*) are species of conservation concern because they are suffering population declines and are keystone species in their ecosystems. Their large area requirements imply that unprotected and ever-increasing agricultural regions can be important habitats as they allow connectivity and dispersal among core protected areas. Yet information on jaguar densities across unprotected landscapes it is still scarce and crucially needed to assist management and range-wide conservation strategies. Our study provides the first jaguar density estimates of Colombia in agricultural regions which included cattle ranching, the main land use in the country, and oil palm cultivation, an increasing land use across the Neotropics. We used camera trapping across two agricultural landscapes located in the Magdalena River valley and in the Colombian llanos (47–53 stations respectively; >2000 trap nights at both sites) and classic and spatially explicit capture-recapture models with the sex of individuals as a covariate. Density estimates were 2.52±0.46–3.15±1.08 adults/100 km^2^ in the Magdalena valley, whereas 1.12±0.13–2.19±0.99 adults/100 km^2^ in the Colombian llanos, depending on analysis used. We suggest that jaguars are able to live across unprotected human-use areas and co-exist with agricultural landscapes including oil-palm plantations if natural areas and riparian habitats persist in the landscape and hunting of both jaguar and prey is limited. In the face of an expanding agriculture across the tropics we recommend land-use planning, adequate incentives, regulations, and good agricultural practices for range-wide jaguar connectivity and survival.

## Introduction

Due to their charisma and functional role in maintaining ecosystem integrity and services [1,2] large carnivores such as the big cats have been a focus of conservation research and action [3]. However, despite conservation efforts, their populations are still declining and their range contracting with important ecological consequences [1,4]. Habitat loss driven by agricultural expansion is the main cause of biodiversity decline globally [5,6] and constitutes a severe threat for large carnivores because they occur at low densities, have slow population growth rates, require large areas and sufficient prey [7–9], all of which make them particularly vulnerable to extinction. Their prey requirements also make them susceptible to conflict with humans and retaliatory killing, further increasing their vulnerability [10,11].

Abundance, density, and distribution estimates are key information for conservation and management strategies, and when they refer to modified areas they can provide valuable information on species tolerance limits [12]. Because of large carnivores’ cryptic nature and large ranges it is inherently difficult to assess their population status, hindering conservation efforts, particularly across unprotected areas. Spatial requirements of large carnivores imply that most protected areas alone are not viable for their survival [13,14] and that they have to be integrated with increasing human modified areas into wider connectivity landscapes [15–18]. There is evidence on the role of unprotected areas for carnivore conservation: species like cheetahs, wolves (*Canis lupus*), pumas (*Puma concolor*), leopards (*Panthera pardus*), and jaguars are able to live in human use landscapes [12,19–21].

The jaguar is the only living representative of the genus Panthera found in the New World and it is the largest cat existing in the Americas [22]. It ranges from Mexico to Argentina and it has been lost from over 50% of its historical range [15]. Jaguars are keystone species [1] and they are considered Near Threatened by the IUCN. They are a species of conservation concern due to habitat loss, poaching of its prey, and retaliatory killing following predation of livestock [23].

As for the other large carnivores, protected areas are too few in number for long-term jaguar conservation, which requires a landscape approach with both protected and unprotected lands [15,24]. However the latter have been neglected, and only 15% (N = 12) of the jaguar population density estimates available [25] refer to areas that are completely unprotected. Therefore it is crucial to obtain more estimates across such areas as agricultural and oil palm (*Elaeis guineensis*) landscapes. The latter are particularly of concern as a driver of impoverished habitat with unknown survival value for jaguars [25, 26, 27,28].

Colombia is extremely important for range-wide jaguar conservation and connectivity due to its position between Central and South America [24]. In Colombia, jaguars inhabit the Amazon and Llanos regions, the Pacific coast, inter-Andean valleys, and the northern area along the Caribbean coast, yet only two jaguar densities estimate are available and they were both in the Amazon [29]. Here we use both SECR and CR models to produce the first jaguar density estimates of Colombia outside the Amazon: across an oil palm landscape in the Magdalena watershed and in an extensive cattle ranch in the llanos ecosystem. These data illustrates the complementary conservation role of unprotected areas to wide ranging large carnivores such as the jaguar.

## Methods

### Ethics statement

The study was conducted across different private properties in the Departments of Santander and Casanare, Colombia. It did not involve manipulation or handling of any living organism as we used non-invasive methods for data collection. Following regulations from the Colombian Government, a study of such nature does not require permits or approval from an Institutional Animal Care and Use Committee or equivalent committee. Each landowner was consulted and personally granted us permission to access and collect data on his or her property.

### Study Areas

**Fig 1:**
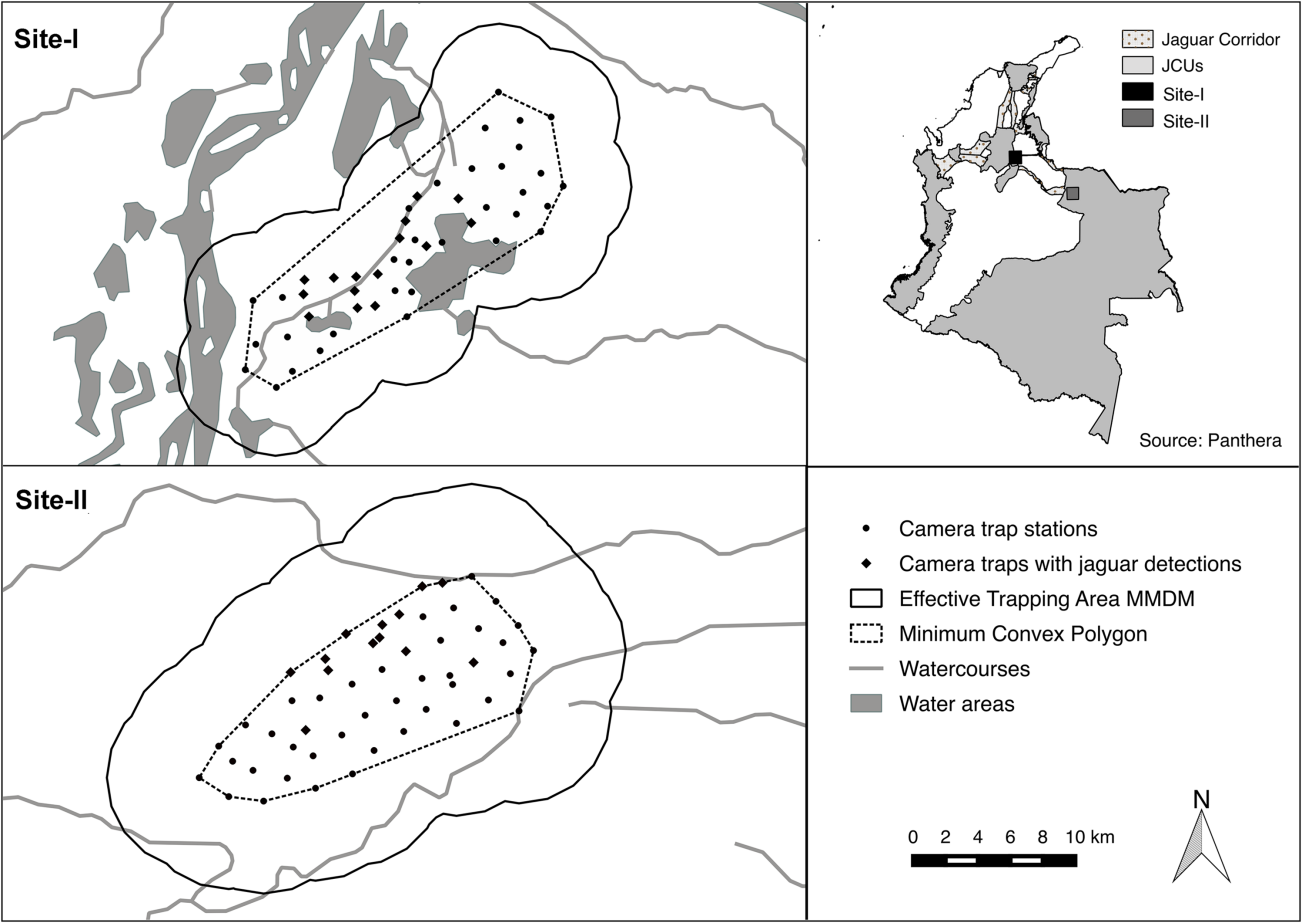
Study areas. Location of the sites in regard to the Jaguar Corridor and Jaguar Conservation Units (JCUs) in Colombia [15,24,33], and map of the study sites with camera locations. Site-1 is part of the Magdalena River valley, while Site-2 is located in the Orinoco River basin. Both sites were surveyed in 2014.

We conducted the study at two sites in Colombia (Fig 1). Site-I is located in the central part of the Magdalena River inter-Andean valley (7.3751981N -73.8841707E to 7.5404397N -73.7117879E) in the Department of Santander. The region is characterized by humid tropical forests and wetlands [30], however most has been converted into cattle ranches and oil-palm plantations while the remaining natural habitats are threatened by further agricultural and oil palm conversion [31,32]. The climate is tropical with mean annual temperature of 27°C and bimodal rainfall of 2100–2600 mm annually [30]. Land tenure consists mainly of private properties; there are no protected areas; and land cover types comprise secondary forest, shrub, wetlands, pastures, crops, oil-palm plantations, and urban areas.

Site-II is located in the Orinoco River basin in the llanos region and in the Department of Casanare (5.9552203N -71.4833672E to 6.0812814N -71.2976157E). This area is naturally characterised by seasonally flooded tropical savannahs bisected by riparian forests, and the dominant land use is extensive cattle ranching with introduced grasses [30]. Mean annual temperature is 27°C and average rainfall is between 1000 and 3000 mm with a very marked wet season between April and November [30]. The area is part of the Llanos Amazon Jaguar Conservation Unit (JCU) [15] (Fig 1) and hosts most of its biodiversity richness along water bodies [34]. Land tenure consists mainly of private properties and land cover types include natural and secondary forest, natural and introduced grasslands, and wetlands.

Jaguar prey species have been historically hunted at both sites and hunting still occurs for subsistence and commercial reasons [35]. Killing of jaguars is rare at Site-I [36] while more frequent at Site-II, although no exact data is available [37], there has been an estimate based on historical records of killings of 1 individual every 250 km^2^ per year [38]. Widespread extensive cattle ranching at Site-II favours the occurrence of jaguar predation on livestock and consequent persecution from ranchers [21,39].

### Camera trapping

Camera trapping surveys were done between April and August 2014 at Site-I and in April-May 2014 at Site-II. We employed a camera design which is recommended for jaguar studies [25,40,41] and meets capture recapture models’ assumptions, i.e. the population is closed and all individuals have at least some probability of being captured [42,43]. We conducted surveys < 120 days and we placed cameras at a distance of 1.6 ± 0.2 km to meet the assumptions of the models.

We employed paired stations and a block design of 47 stations at Site-I, covering an area of 154.8 km^2^ (minimum convex polygon), while a continuous design and 53 station across 151.3 km^2^ at Site-II (Fig 1). We used Cuddeback Attack and Ambush, and Panthera series 3 and 4 cameras and set them at a height of 35 cm. Paired stations ensure photographs of both flanks of each passing individual for complete identification purposes.

### Data processing and capture-recapture analysis

Jaguar individuals were identified from their spot and rosette patterns and sexed by visual inspection of external genitalia. We then produced adult density estimates using both SECR and conventional CR. SECR models were applied to jaguars for the first time by Sollmann et al. [44] and have the advantage of not requiring arbitrary buffers to estimate the Effective Trapping Areas (ETAs) and hence density values [45,46]. They use the individuals’ spatial locations to determine their activity centres or home range centres and then estimate the density of home range centres across a polygon which contains the trap grid [45,46]. SECR models also assume that home ranges are circular and stable during the survey, individuals activity centres are randomly distributed (as a Poisson process), and the encounter rate of an individual with a trap decreases with increasing distance from the activity centre following a predefined function [45,46].

The most commonly used function and the one we also used is the half-normal detection function, which describes the probability of capture (P) of an individual i at a trap j as a function of distance (d) from the activity centre of the individual to the trap as follow: Pij = g0 exp (- dij^2^/(2σ^2^), where g0 is the probability of capture when the trap is located exactly at the centre of the home range, and sigma (σ) is a spatial parameter related to home range size [45]. One model that is most relevant to camera trapping studies is the Bernoulli or binomial encounter model, under which an individual can be recorded at different camera stations during one sampling occasion but only once at each station [41,47]. The models can be fitted in a maximum-likelihood framework [48,49] or in a Bayesian framework using data augmentation [46,50]. We chose maximum likelihood because it gives comparable results to the Bayesian framework [25,51] with quicker computation times and used the package secr in R [52].

We included the exact number of days that each station was active and allowed both parameters g0 and σ to vary with sex of the individuals [25,44,53]. We compared four models using the Akaike Information Criterion (AIC) [54]: “SECR.0” (null model), “SECR.sex.g0” (g0 varies between males and females), “SECR.sex.σ” (σ varies between males and females), and “SECR.sex” (both g0 and σ vary between males and females). Including individuals sex as a covariate is important because jaguar populations have unequal ranging patterns between sexes, which would affect capture probabilities [40,43,55].

For non-spatial capture recapture analysis we converted the capture histories of each individual into a 1 and 0 matrix and we grouped 6 survey days into one sampling occasion [41,44]. Following we analysed the data with the full likelihood closed captures models in program MARK [56] and compared three models that differ in assumed sources of variation in capture probability (p) using AIC: “Mo” (null model), “Mh” (p varies between individuals), and “Msex” (p varies between males and females). Following, we estimated the effective trapping areas by adding a buffer to the cameras polygon equal to the Mean Maximum Distance Moved (MMDM). The MMDM is calculated by taking the average of the maximum distances between capture locations for all individuals [55]. Finally we calculated density as: D = N/ETA. We further included densities estimated with program Capture, the Jacknife estimator and both MMDM and 1/2MMDM in S1 Appendix.

### Prey capture rates

We calculated capture rates for jaguar prey species at the two sites using the total number of independent capture events of each species divided by the number of trap-nights and expressed as records per 100 trap nights [57,58]. Independent capture events were defined as consecutive photographs of individuals of the same species taken more than 12 hrs apart for gregarious species (i.e. capybaras, *Hydrochoerus sp*.; collared peccaries, *Pecari tajacu*; and white-tailed deer, *Odocoileus virginianus)*, and more than 30 min apart for all other species [58]. A species was considered prey if reported in jaguar diet studies [39,59–61]. It is debatable whether capture rates actually reflect abundance [57,62] hence we do not report them to make inferences about population sizes but for descriptive purposes.

## Results

We recorded seven females (49 events) and three males (39 events) at Site-I and two females (8 events), three males (57 events), and one adult individual of unknown sex at Site-II (Table 1). Four of ten individuals recorded at Site-I have been recorded in the area since 2012. The average number of captures per individual was lower for females than males at both sites: 7 (1–13) vs. 13 (3–26) at Site-I and 4 (3–5) vs. 19 (12–28) at Site-II. Captures of multiple individuals at the same camera stations were common and up to six individuals were recorded at one station in Site-I.

**Table 1:** Parameters and survey features for Site-I and Site-II.

	Site-I	Site-II
Location	Magdalena River valley	Orinoco River basin
Survey period	April-August 2014	April-May 2014
Traps active	47	52
Trap nights	2251	2457
Minimum Convex Camera polygon (km2)	154.8	151.3
N recorded	10	6
MMDM (km)	4.2	5.7
Effective sampled area (km^2^)	396.2	537.2

N = Number of individuals; MMDM = Mean Maximum Distance Moved

The best CR model for Site-I was Mh (AIC = 130.2), but M0 (AIC = 130.6) was also strongly supported (ΔAIC<2); whereas for Site-II the best CR model was Msex (AIC = 75.4) followed by Mh (AIC = 76.6), which also had strong support (ΔAIC<2) (Table 2). Both supported CR models estimated N = 10.00 ± 0.00 (SE) and density = 2.52 ± 0.46 (95% CI: 1.63–3.42) (N/100 km^2^) at Site-I while N = 6.00 ± 0.00 (6.00–6.00) and density = 1.12 ± 0.13 (95% CI: 0.86–1.38) (N/100 km^2^) at Site-II.

**Table 2:** Model selection parameters for both Capture-Recapture (CR) and Spatially Explicit Capture Recapture (SECR) models at Site-I and Site-II.

Site-I Magdalena River valley						Site-II Orinoco River basin					
	AIC	ΔAIC	W	K	Dev.		AIC	ΔAIC	W	K	Dev.
CR Mh	130.2	0	0.55	2	108.4	CR Msex	75.4	0	0.60	2	65.4
CR M0	130.6	0.4	0.45	1	112.7	CR Mh	76.6	1.2	0.33	3	62.2
CR Msex	138.8	8.6	0.00	3	121.6	CR M0	79.7	4.1	0.07	1	69.4
SECR.sex.g0	924.2	0	0.66	5	894.2	SECR.sex	612.5	0	0.66	6	588.5
SECR.sex	925.6	1.4	0.34	5	893.6	SECR.sex.g0	614.0	1.5	0.32	5	592.0
SECR.sex.σ	937.3	13.1	0.00	6	907.3	SECR.sex.σ	619.8	7.3	0.02	5	597.8
SECR.0	953.4	29.2	0.00	4	925.4	SECR.0	628.7	16.2	0.00	4	608.7

AIC = Akaike Information Criterion; ΔAIC = difference in AIC values between each model and the model with the lowest AIC; W = AIC model weights; K = number of model parameters; Dev. = Model Deviances. Mh: capture probability varies between individuals; M0: null model, Msex: capture probability varies between males and females. g0 = probability of capture at the home range centre, σ = spatial parameter related to home range size; SECR.sex.g0: g0 varies between males and females; SECR.sex: both g0 andσ vary between males and females; SECR.sex.σ:σ varies between males and females; SECR.0: null model.

The best SECR model (AIC = 924.2) for Site-I allowed g0 to vary with sex but had a fixed σ (SECR.sex.g0), while for Site-II the best model (AIC = 612.5) allowed both parameters to vary with sex (SECR.sex). However, SECR.sex and SECR.sex.g0 also had strong support (ΔAIC<2) for Site-I and Site-II respectively (Table 2).

Therefore we report density estimates and parameters for both SECR models at both sites (Table 3). Under the secr.sex model g0 resulted much lower for females at both sites (0.051 vs. 0.813 at Site-I; 0.009 vs. 0.118 at Site-II), whereas σ was smaller for females at Site-I while for males at Site II (Table 3). This led to females home ranges estimates of 42.7 km^2^ and 102.1 km^2^ at Site-I and Site-II respectively, and to male home range estimates of 52.8 km^2^ at Site-I and 38.3 km^2^ at Site-II

**Table 3:** Density and parameters estimated by the two best Spatially Explicit Capture Recapture (SECR) models, i.e. SECR.sex and SECR.sex.g0, at Site-I and Site-II.

	Site-I Magdalena River valley					Site-II Orinoco River basin				
	Value	SE	95% LCI	95% UCI	CV	Value	SE	95% LCI	95% UCI	CV
g0 females SECR.sex	0.051	0.020	0.024	0.106	39%	0.009	0.005	0.003	0.024	56%
g0 males SECR.sex	0.813	0.556	0.003	1.000	68%	0.118	0.025	0.077	0.176	21%
σ females (km) SECR.sex	1.507	0.147	1.245	1.822	10%	2.327	0.693	1.315	4119	30%
σ males (km) SECR.sex	1.674	0.174	1.366	2.051	10%	1.426	0.129	1.195	1.701	9%
**D (N/100km**^**2**^**) SECR.sex**	**3.15**	**1.08**	**1.64**	**6.05**	**34%**	**1.88**	**0.87**	**0.79**	**4.48**	**46%**
g0 females SECR.sex.g0	0.046	0.016	0.023	0.088	35%	0.013	0.006	0.006	0.030	46%
g0 males SECR.secr.g0	0.999	0.000	0.999	0.999	0%	0.108	0.022	0.071	0.159	20%
σ (km) SECR.sex.g0	1.617	0.042	1.537	1.701	3%	1533	133	129	1818	9%
**D (N/100km**^**2**^**) SECR.sex.g0**	**3.04**	**1.02**	**1.60**	**5.78**	**34%**	**2.19**	**0.99**	**0.93**	**5.13**	**45%**

SE = Standard error; LCI and UCI = lower and upper confidence intervals respectively; CV = Coefficient of Variation; D = Density. Density values are in bold. g0 = probability of capture at the home range centre, σ = spatial parameter related to home range size; SECR.sex.g0: g0 varies between males and females; SECR.sex: both g0 and σ vary between males and females; SECR.sex.σ: only σ varies between males and females; SECR.0: null model.

We recorded 12 prey species at Site-I and 16 at Site-II with Central American agouti (*Dasyprocta punctuata*) and black agouti (*Dasyprocta fuliginosa*) being the most frequently captured species at Site-I and 2 respectively (S2 Appendix).

## Discussion

It has been recognised that protected areas are inadequate for the long-term conservation of jaguars [18,22]. Therefore, estimating their population size and density in increasingly modified landscapes helps understanding the extent to which jaguar can persist in human areas and informs conservation planning. We provided the first jaguar density estimates of Colombia outside of the Amazon forest [29] and in agricultural landscapes. Cattle ranching is the primary land use in the country and oil palm cultivation is an emerging land use across the Neotropics [28,63].

### Jaguar densities

Our results at both sites show that unprotected and productive areas with remaining natural habitats can be important for jaguar populations. Protected areas should always be considered core refuges and they can have a direct effect on population size [29], but large-scale landscape connectivity is also essential. National Parks such as Iguazu and Emas can only harbour small jaguar populations if surrounded by matrices of converted habitat and poaching, and jaguar densities were estimated as low as 0.5–0.9 and 0.3 at those parks respectively [44,64].

Jaguar densities tend to be greater in wetter and prey-rich habitats such as lowland tropical forests [40,53,65] or in the flooded plains of the Pantanal [66] and lower in drier habitats such as the Gran Chaco [67] and Cerrado [44] (S3 Appendix). Densities are also affected by the level of human use: they can be high in productive lands such as cattle ranches in the Pantanal [66], and forestry concessions in the Cerrado [68] and the Amazon [53], but they become low across highly degraded habitats such as Brazilian Atlantic forest [64] or heavily hunted regions [69].

Site-I is within the tropical forest biome and has abundant wetlands and seasonal flooded areas [30], hence it is part of the wetter habitats of the jaguar range. However the SECR density values we obtained at the site (3.0±1.0–3.1±1.1) are lower than similar habitats (S3 Appendix). Tobler et al. [53] report an average jaguar density of 4.4 ± 0.7 across the South Western Amazon when using SECR models, while in the Pantanal densities were estimated as high as 6.7 ± 1.1 using a reliable buffer obtained with telemetry [66]. Our lower estimates may have resulted from much of the region being converted to agriculture, including oil palm plantations. However, they are higher than we expected given the extensive habitat conversion. These densities may have resulted from remaining wetlands and existing connectivity with the San Lucas JCU towards the West of the study area as a source for the population (Fig 1). The importance of wetlands for jaguars in the study area is further confirmed by the fact that jaguar were recorded mainly at camera stations situated in wetland habitats and never in oil palm habitats. Connectivity between this area and the Catatumbo and Llanos-Amazon JCUs towards the East and South East (Fig 1) is uncertain and should be assessed.

Carnivore densities are highly dependent on the prey base available [9,70] and levels of hunting of both prey and carnivores themselves [69]. Killing of jaguars at Site-I is rare [36] but larger prey species such as deer, tapirs *(Tapitus terrestris)*, peccaries, giant anteaters (*Mymecophaga tridactyla*), and capybaras on which jaguar depend in other regions [59–61] were absent or infrequent, likely due to both habitat loss and hunting. These species are regularly hunted for subsistence and commercial purposes in Colombia [35]. It is therefore possible that jaguars complement their terrestrial prey base with aquatic species such as caimans (*Caiman crocodilus*) and turtles (*Podocnemis* and *Trachemys sp*) as found elsewhere [71].

Site-II is part of the Llanos-Amazon JCU (Fig 1), indicating that jaguars at this site are part of a larger population in a connectivity landscape. The llanos’ biome (i.e. seasonally flooded grasslands [30]) is similar to the Pantanal but with some important differences. There is more prey biomass in the Pantanal [72] and flooding is one quarter of the year longer than in the llanos, thus limiting productive human land use. Furthermore, the llanos also were colonized 200 years earlier than the Pantanal and display much higher human density and hunting levels. Finally, jaguar densities in the Pantanal were estimated across ranches without hunting in the past 15 years and with extremely low human density. All these factors could explain the lower jaguar density (1.9 ± 0.9–2.2 ± 1.0) we obtained.

Lower jaguar numbers in the llanos could also be due to retaliatory killing following livestock predation. Incidents of jaguar predation on livestock do occur [37,73] however, currently there is a paucity of data regarding human persecution of jaguar. Past systematic hunting of jaguars for the spotted pelt trade could also explain low population numbers [74] but again, that would assume little to no recovery.

Usually more males than females are recorded in camera trap studies because males tend to move more and have larger home ranges [75]. This is in accordance to what we obtained at Site-II, however the sex ratio was skewed to females (2.3:1) at Site-I, where we even recorded mating events and cubs. This, in addition to recording resident jaguars (since 2012), suggests that the area is important for jaguar conservation and possibly constitutes a breeding refuge [75].

### Methodological considerations and sex specific parameters

Our survey effort (47–53 camera stations) was more comprehensive than most jaguar studies, as only 15% of jaguar studies reviewed by Tobler and Powell [25] used > 40 camera stations. Density estimates become unbiased and precision increases if the camera polygon is asymmetrical [76] and encompasses several home ranges [25,77] which is logistically challenging when sampling wide- ranging species like jaguars. However, even if we assume large home ranges (400 km^2^) and low detection probabilities at home range center (g0 = 0.01) the density bias for polygons like ours, ca. 150km^2^, is less than 10% [25].

Jaguar home ranges in wetter habitats vary greatly: some studies [78–81] estimated home ranges size smaller or comparable to what we obtained at Site-I, while others have reported them much larger [53,82–84]. At Site-II, female home range was larger than reported by Scognamillo et al. [85] in the Venezuelan Llanos (53–83 km^2^), whereas for males it was the opposite. Female home ranges are usually smaller than those of males’ [53,80,83]. We observed the opposite pattern at Site-II and could be an artefact of sample size. SECR models assume circular home ranges, and that may have been violated in our landscapes where jaguars move along watercourses and riparian galleries.

Because of sex-specific detection probabilities and home range sizes, including sex as a covariate reduces the bias in density estimates and produced better SECR models at both sites. However the best CR models at Site-1 did not include sex as a covariate and it could be because CR models do not include spatial behaviour, hence reducing differences between the sexes. Ultimately, with small sample sizes, partitioning the data into sex specific group is a trade-off between bias and precision. We also recommend larger camera polygons than ours to increase the number of individuals captured and achieve more accurate density estimates.

We concur with other authors [25,44,53], and recommend using SECR models over CR ones when estimating densities because they are not biased by arbitrary buffers, are robust even with smaller grids [76], and can account for larger numbers of individual and site based covariates, producing more reliable estimates and addressing many issues outlined by [86]. Obtaining reliable and comparable estimates is key to avoid biased population statuses, underestimation of threats, and delayed conservation interventions, exposing the species at greater risk of decline. Lastly, we may have under-detected some prey species as all our cameras were placed on roads and trails and might have ignored micro-habitats that are important for certain prey species, however placing cameras on trails is still considered the best option to optimize detection of multiple (forest) mammals at once [57,87,88].

## Conclusion

In the case of wide-ranging species such as large carnivores, human-use areas are important habitats for connectivity and dispersal between core protected areas as well as for resident and breeding populations [12,19,89]. Therefore it is essential to study these species in unprotected and modified areas to understand the limits to their tolerance and survival [12]. Our results provide additional evidence on the role of unprotected areas for carnivore conservation, advance current understanding of jaguars in agricultural areas, and provide the first jaguar density estimates in both the llanos ecosystem and in an oil palm landscape. They also indicate that productive areas with extensive cattle ranching and oil palm cultivation can be important for jaguar conservation as long as natural habitats such as wetlands, forests, and riparian galleries persist in the landscape. Natural areas in human-dominated regions are crucial for the survival of landscape species worldwide allowing them to disperse and thrive beyond protected areas [15–17,90].

As agriculture and oil palm cultivation continue to expand across the tropics they need to be integrated into range-wide jaguar conservation strategies. For long-term jaguar conservation it is key to engage landowners, implement land-use plans in both regions to maintain natural habitats in the landscape, and establishing further oil palm plantations in already disturbed areas, as identified by García-Ulloa et al. [91]. Across cattle ranching regions it is also crucial to adopt optimal livestock management practices to ensure low predation and low levels of human-jaguar conflict [92,93].

## Supporting Information

S1 Appendix Density results Capture & Mh. (DOCX)

S2 Appendix Independent capture events and capture rates of jaguars and their prey species at both sites. (DOCX)

S3 Appendix Jaguar (*Panthera onca*) density estimates from camera trap surveys, modified from Tobler and Powell (2013). (DOCX)

S4 Appendix Data, Site-I. (DOCX)

S5 Appendix Data, Site-II. (DOCX)
