# Supplementary material for: Jaguar Densities across Human-Dominated Landscapes in Colombia: The Contribution of Unprotected Areas to Long Term Conservation
Source: PLoS One. 2016 May 4;11(5):e0153973. doi: 10.1371/journal.pone.0153973 (PMC4856405; doi:10.1371/journal.pone.0153973)
Supplement: S1 Appendix — (DOCX) [file pone.0153973.s001.docx]

**S1 Appendix. Density results obtained with Capture & Mh.**

|  | **Site 1** | | | | | **Site 2** | | | | |
| --- | --- | --- | --- | --- | --- | --- | --- | --- | --- | --- |
|  | **Value** | **SE** | **95% LCI** | **95% UCI** | **CV** | **Value** | **SE** | **95% LCI** | **95%**  **UCI** | **CV** |
| N (Mh) | 11 | 1.42 | 11 | 18 | 13% | 7 | 1.38 | 7 | 13 | 20% |
| Average capture probability (Mh) | 0.34 |  |  |  |  | 0.39 |  |  |  |  |
| MMDM (km) | 4.2 |  |  |  |  | 5.7 |  |  |  |  |
| Effective sampled area MMDM (km^2^) | 396.2 |  |  |  |  | 537.2 |  |  |  |  |
| ½ MMDM (km) | 2.1 |  |  |  |  | 2.9 |  |  |  |  |
| Effective sampled area ½MMDM (km^2^) | 225.1 |  |  |  |  | 308.1 |  |  |  |  |
| D Mh MMDM (N/100km^2^) | **2.77** | 0.66 | 2.77 | 4.54 | 24% | **1.30** | 0.30 | 1.30 | 2.41 | 23% |
| D Mh ½MMDM (N/100km^2^) | **4.88** | 1.32 | 4.88 | 8.00 | 27% | **2.27** | 0.48 | 2.27 | 4.22 | 21% |
