# Supplementary material for: Jaguar Densities across Human-Dominated Landscapes in Colombia: The Contribution of Unprotected Areas to Long Term Conservation
Source: PLoS One. 2016 May 4;11(5):e0153973. doi: 10.1371/journal.pone.0153973 (PMC4856405; doi:10.1371/journal.pone.0153973)
Supplement: S2 Appendix — (DOCX) [file pone.0153973.s002.docx]

**S2 Appendix. Independent capture events and capture rates of jaguars and their prey species at both sites.**

|  | **Site-I** | | **Site-II** | |
| --- | --- | --- | --- | --- |
|  | **Capture**  **events** | **Capture**  **rates** | **Capture**  **events** | **Capture**  **rates** |
| ***Panthera onca*** | 111 | 4.93 | 76 | 3.09 |
| *Caiman crocodilus* | - | - | 5 | 0.20 |
| *Cebus albifrons* | 40 | 1.78 | - | - |
| *Chelonoidis denticulata* | - | - | 4 | 0.16 |
| *Cuniculus paca* | 15 | 0.67 | 51 | 2.08 |
| *Dasyprocta fuliginosa* | - | - | 278 | 11.31 |
| *Dasyprocta punctata* | 121 | 5.38 | - | - |
| *Dasypus novemcinctus* | 7 | 0.31 | 16 | 0.65 |
| *Didelphis marsupialis* | 9 | 0.40 | 18 | 0.73 |
| *Hydrochoerus hydrochaeris* | - | - | 238 | 9.68 |
| *Hydrochoerus isthmus* | 7 | 0.31 | - | - |
| *Iguana iguana* | 4 | 0.18 | 13 | 0.53 |
| *Mazama americana* | - | - | 52 | 2.12 |
| *Myrmecophaga tridactyla* | 6 | 0.27 | 199 | 8.10 |
| *Odocoileus virginianus* | - | - | 161 | 6.55 |
| *Pecari tajacu* | 5 | 0.22 | 93 | 3.79 |
| *Philander opossum* | - | - | 1 | 0.04 |
| *Procyon cancrivorus* | 60 | 2.67 | 1 | 0.04 |
| *Tamandua tetradactyla* | 6 | 0.27 | 33 | 1.34 |
| *Tupinambis sp.* | 4 | 0.18 | 2 | 0.08 |
