# Supplementary material for: Jaguar Densities across Human-Dominated Landscapes in Colombia: The Contribution of Unprotected Areas to Long Term Conservation
Source: PLoS One. 2016 May 4;11(5):e0153973. doi: 10.1371/journal.pone.0153973 (PMC4856405; doi:10.1371/journal.pone.0153973)
Supplement: S3 Appendix — (DOCX) [file pone.0153973.s003.docx]

**S3 Appendix. Jaguar (*Panthera onca*) density estimates from camera trap surveys, modified from Tobler and Powell (2013).**

Densities are based on the Mh model and a buffer of ½ MMDM, which was the most commonly used method. When available, density estimates based on other methods are also reported. We report average densities when more estimates were available for the same areas and highlighted rows with density estimates that correspond to exclusively unprotected areas. Mh assumes that capture probability varies across individuals. MMDM=Mean Maximum Distance Moved.

^a^ Density estimates based on the Mh MMDM method

^b^ Density estimates based on a spatially explicate capture-recapture (SECR) model

^c^ Approximate density estimate not based on capture-recapture method

^d^ Density estimate based on the Barker robust design model and MMDM

| ***Country*** | ***Survey*** | ***Density Mh ½ MMDM (Ind. 100 km-2)*** | ***Other density estimates (Ind. 100 km^-2^)*** | ***Biome/Habitat*** | ***Protected area*** | ***Source*** |
| --- | --- | --- | --- | --- | --- | --- |
| Argentina | Iguazu 2004-2006 | 1.26 ±0.34 | 0.49±0.16^a^ | Semi deciduous forest | National Park and Forestry Reserve | Paviolo et al. 2008 |
| Argentina | Urugua-i | 0.3^c^ | 0.12^a^ | Semi deciduous forest | Provincial Park and Private Reserve | Paviolo et al. 2008 |
| Argentina | Yaboti | 0.2^c^ | 0.11^a^ | Semi deciduous forest | Forestry Reserve | Paviolo et al. 2008 |
| Belize | Cockcomb basin | 10.50 ±2.13 |  | Broadleaf tropical moist rainforest | Wildlife Sanctuary | Silver et al. 2004; unknown in Maffei et al. 2011; Harmsen 2006 |
| Belize | Chiquibul | 7.48±2.74 |  | Mosaic of deciduous semi-evergreen and deciduous seasonal forest. | Forest Reserve and National Park | Silver et al. 2004 |
| Belize | Fireburn | 5.31±1.76 |  | Tropical moist lowland forests | Private Reserve, Mesoamerican Biological Corridor | Miller 2006 |
| Belize | Gallon Jug Estate 2004-2005 | 10.05±1.71 |  | Tropical moist lowland forests | Private protected area | Miller 2005 |
| Belize | Mountain Pine Ridge | 3.81 |  | Tropical pine forests | Forest Reserve | M. Kelly unpubl. Data in Maffei et al. 2011 |
| Bolivia | Cerro Cortado I-II Kaa-Iya | 5.20±1.42 |  | Xeric Chacoan forest | National Park and Indigenous communal lands | Silver et al. 2004; Maffei et al. 2004 |
| Bolivia | El Encanto | 5.66±2.33 |  | Cerrado / tropical dry forests (Chiquitano dry forest) | Certified forestry concession | Arispe et al. 2007 |
| Bolivia | Estacion Isoso I-II, Kaa-Iya 2005-2006 | 3.54±0.60 |  | Chaco / tropical dry forests (transitional Chaco-Amazon) | National Park | Maffei et al. 2006; Romero-Munoz et al. 2007 |
| Bolivia | Guanaco, Kaa-Iya I-II | 2.07±0.28 |  | Chaco / tropical dry forests (grasslands) | National Park and cattle ranches | Cuellar et al. 2004a; Cuellar et al. 2004b |
| Bolivia | Palmar I-II, Kaa-Iya | 1.22±0.06 |  | Chaco / tropical dry forests (transitional Chaco-Chiquitano) | National Park, private reserve, and cattle ranch | Romero-Munoz et al. 2007; Montano et al. 2007 |
| Bolivia | Ravelo I-II, Kaa-Iya | 1.92±0.45 |  | Chaco / tropical dry forests (transitional Chaco-Chiquitano) | National Park | Cuellar et al 2003; Maffei et al. 2004 |
| Bolivia | Rios Tuichi and Hondo, Madidi | 2.26±0.97 |  | Tropical Andes / tropical moist lowland forest | National Park | Wallace et al. 2003; Silver et al. 2004 |
| Bolivia | San Miguelito | 7.61±1.43 |  | Cerrado / tropical dry forests (Chiquitano dry forest) | Private reserve and cattle ranch | Rumiz et al. 2003; Arispe et al. 2005 |
| Bolivia | Tucavaca I-II, Kaa-Iya | 2.83±0.52 |  | Chaco / tropical dry forests (transitional Chaco-Chiquitano) | National Park | Silver et al. 2004;Maffei et al. 2004 |
| Brazil | Emas National Park | 2 |  | Cerrado / tropical dry forests | National Park | Silveira 2004 |
| Brazil | Emas National Park | - | 0.51±019^a^ 0.29±0.10^b^ | Cerrado / tropical dry forests | National Park | Sollmann et al. 2011 |
| Brazil | Fazenda Santa Fe | 2.59±1.03 |  | Amazon / tropical moist forests – Cerrado / tropical dry forests ecotone | Cattle ranch, State Park | L. Silveira and N.M. Negroes in Maffei et al. 2011 |
| Brazil | Fazenda Sete 2003-2004 | 11.00±1.23 | 5.75±0.81^a^ | Pantanal / herbaceous lowland grasslands | Cattle ranch | Soisalo and Cavalcanti 2006 |
| Brazil | Moro do Diablo | 2.47±0.46 |  | Atlantic / tropical moist lowland forest | National Park | Cullen 2006 |
| Brazil | Serra da Capivara | 2.67±1.06 | 1.28±0.62^a^ | Caatinga/xerics | National Park | Silveira et al. 2010 |
| Colombia | Amacayacu | 4.2 |  | Amazon / tropical moist lowland forest | National Park and indigenous territory | Payan 2009 |
| Colombia | Calderon river valley | 2.5 |  | Amazon / tropical moist lowland forest | National Forestry Reserve and indigenous territory | Payan 2009 |
| Costa Rica | Corcovado | 6.98±2.36 |  | Tropical moist lowland forest | National Park | Salom-Perez et al. 2007 |
| Costa Rica | Golfo Dulce / Golfito | 2±1.49 |  | Tropical moist lowland forest | Private ranches, Forest Reserve, Wildlife Reserve | Bustamante 2008 |
| Costa Rica | San Cristobal | 6.7 |  | Tropical Rainforest, Low Montane and Premontane Rainforest | Biological; National Park corridor | Rojas 2006 |
| Costa Rica | Talamanca | 1.34±0.48 |  |  |  | Gutierez and Porras 2008 |
| Costa Rica | Talamanca ZPLT (Coton) | 5.42±2.3 | 2.25^a^ | Tropical forest | Protected area | Gonzales-Maya 2007 |
| Ecuador | Yasuni-Waorani | 1.38±0.6 |  | Amazon / tropical moist lowland forest | National Park and indigenous territory | S. Espinoza unpubl. Data in Maffei et al. 2011 |
| Ecuador | Yasuni ITT | 2.2 |  |  | National Park | Araguillin et al. 2010 |
| French Guiana | Counami Forest | 3.3 |  | Amazon / tropical moist lowland forest | Unprotected | Association Kwata 2009 |
| French Guiana | Montagne de Fer | 4.9 |  |  |  | Association Kwata 2009 |
| Guatemala | Carmelita-AFISAP | 11.28±3.51 |  | Tropical moist lowland forest | Forestry concessions | Moreira et al. 2008a |
| Guatemala | La Gloria-Lechugal | 1.54±0.85 |  | Tropical moist lowland forest | Forestry concession, multiple use zone | Moreira et al. 2007 |
| Guatemala | Mirador, Oeste | 1.99±1.57 | 0.9±0.48^a^ |  |  | Moreira et al. 2005 |
| Guatemala | Dos Lagunas Rio Azul | 11.14±7.45 | 7.02±6.44^a^ | Tropical moist lowland forest | National Park | Moreira et al. 2008b |
| Guatemala | Tikal | 6.63±2.46 | 3.39^a^ | Tropical moist lowland forest | National Park | Garcia et al. 2006 |
| Guatemala | Melchor de Mecos | 6.04±1.68 | 2.91±0.72^a^ | Subtropical Humid Forest. | Community concession forest | Moreira et al. 2010 |
| Guatemala | Laguna del Tigre | 6.32±1.66 | 3.73±0.49^a^ | Wetlands, lowland forest | National Park, Concession area | Moreira et al. 2009 |
| Honduras | La Mosquitia | 5.2^c^ |  | Tropical moist lowland forest | Indigenous territory | Portillo Reyes and Hernandez 2011 |
| Mexico | Sonora | 1±1.3^c^ |  | Mexican xerics / tropical thorn scrub | Private Reserve and cattle ranches | Rosas-Rosas 2006 |
| Mexico | San Luis Potosi 2008 | 3.2±1.9 | 1.55±1.93^a^ | Tropical forest, deciduous and evergreen forest | Unprotected | Avila Najera 2009 |
| Mexico | Quintana Roo | 3.88±0.70 | 1.95±0.45^c^ | Tropical forest | Ecological Reserve | Avila Najera et al. 2015 |
| Mexico | Sonora |  | 1.87±0.47 (Barker robust design + MMDM) | Desert scrub, Tropical decisuous forest, grassland | Northern Jaguar Reserve and cattle ranches | Gutierrez Gonzalez et al. 2015 |
| Panama | Darien | 3.12 | 1.70^a^ | Tropical moist lowland forest | National Park | Moreno 2006 |
| Peru | Los Amigos 2005-2007 | 10.11±1.28 | 4.25±0.95^b^ | Tropical Andes / tropical moist lowland forest | Conservation concession | Tobler et al. 2013 |
| Peru | Bahuaja Sonene, Tambopata | 8.1±3.6 |  | Tropical Andes / tropical moist lowland forest | National Parks | Tobler et al. 2013 |
| Peru | Espinoza | 6.9±1.3 | 4.9±1.0^b^ | Tropical Andes / tropical moist lowland forest | Forestry concessions | Tobler et al. 2013 |

Araguillin, E., G. Z. Ríos, V. Utreras, and A. Noss. 2010. Muestreo con trampas fotográficas de mamíferos medianos, grandes y de aves en el Bloque Ishpingo Tambococha Tiputini (ITT), sector Varadero (Parque Nacional Yasuní). Wildlife Conservation Society Ecuador, Quito, Ecuador.

Arispe, R., D. Rumiz, and C. Venegas. 2005. Segundo censo de jaguares (Panthera onca) y otros mamíferos con trampas-cámara en la estancia San Miguelito, Santa Cruz, Bolivia. Wildlife Conservation Society, Santa Cruz, Bolivia.

Arispe, R., D. Rumiz, and C. Venegas. 2007. Censo de jaguares (Panthera onca) y otros mamíferos con trampas cámara en la Concesión Forestal El Encanto. Wildlife Conservation Society, Santa Cruz, Bolivia.

Association Kwata. 2009. Camera-traps for survey of felids in French Guiana 2007-2008. Association Kwata, Cayenne, French Guiana.

Avila Nájera, D. M. 2009. Abundancia del Jaguar (Panthera onca) y de sus Presas en el Municipio de Tamasopo, San Luis Potosí. M.Sc. Thesis. Instituto de Enseñanzas e Investigacion en Ciencias Agricolas, Montecillo, Mexico.

Avila Nájera, D. M, Chavez, C., Lazcano-Barrero, M., Perez-Elizalde, S., ALcantara-Carbajal, J.L. 2015. Population estimates and conservation of felids (Carnivora: Felidae) in Northern Quintana Roo, Mexico. Revista de Biología Tropical/International Journal of Tropical Biology and Conservation 63: 799-813.

Bustamante, A. H. 2008. Densidad y uso de hábitat por los felinos en la parte sureste del área de amortiguamiento del Parque Nacional Corcovado, Península de Osa, Costa Rica. M.Sc. Thesis. Universidad Nacional, Heredia, Costa Rica.

Cuéllar, E., T. Dosapei, R. Peña, and A. Noss. 2003. Jaguar and other mammal camera trap survey Ravelo II, Ravelo field camp (19° 17’ 44” S, 60° 37’ 10” w) Kaa-Iya del Gran Chaco National Park. 18 September - 18 November 2003. Wildlife Conservation Society, Santa Cruz, Bolivia.

Cuéllar, E., J. Segundo, G. Castro, J. Barrientos, Juliet Healy, A. Hesse, and A. Noss. 2004a. Jaguar and other mammal camera trap survey Guanaco area (20° 03’ 03” s, 62° 26’ 04” w) Kaa-Iya del Gran Chaco National Park. 19 December 2003– 16 February 2004. Wildlife Conservation Society, Santa Cruz, Bolivia.

Cuéllar, E., J. Segundo, G. Castro, A. Segundo, A. Hesse, and A. Noss. 2004b. Jaguar and other mammal camera trap survey Guanaco II (20° 03’ 03” S, 62° 26’ 04” W) Kaa-Iya del Gran Chaco National Park. 18 August - 18 October 2004. Wildlife Conservation Society, Santa Cruz, Bolivia.

Cullen, L. 2006. Jaguars as landscape detectives for the conservation of Atlantic Forest in Brazil. Univerity of Kent, Canterbury, UK.

Foster, R. 2008. The ecology of jaguars (Panthera onca) in a human-influenced landscape. Ph.D. Dissertation. University of Southampton, Southampton, UK.

García, R., R. B. McNab, J. S. Shoender, J. Radachowsky, J. Moreira, C. Estrada, V. Méndez, D. Juárez, T. Dubón, M. Córdova, F. Córdova, F. Oliva, G. Tut, K. Tut, E. González, E. Muñoz, L. Morales, and L. Flores. 2006. Los jaguares del corazón del Parque Nacional Tikal, Petén, Guatemala. Wildlife Conservation Society-Programa para Guatemala, Guatemala.

Gonzáles-Maya, J. F. 2007. Densidad, uso de hábitat y presas del jaguar (*Panthera onca*) y el conflicto con humanos en la región de Talamanca, Costa Rica. M.Sc. Thesis. Centro Agronomico Tropical de Investigación y Enseñanza, Turrialba, Costa Rica.

Gutiérez, D. C. and J. C. Porras. 2008. Ecolgía poblacional de jaguar (*Panthera onca*) y puma (*Puman concolor*) y dieta de jaguar, en el sector Pacífico de la Cordillera de Talamanca, Costa Rica. B.Sc. Thesis. Universidad Latina de Costa Rica, San José, Costa Rica.

Gutiérrez-González, C.E., Gómez-Ramírez, M.A., López-González, C.A., Doherty, P.F. Jr. 2015. Are Private Reserves Effective for Jaguar Conservation? PLoS ONE 10(9): e0137541. doi:10.1371/journal.pone.0137541

Harmsen, B. J. 2006. The use of camera traps for estimating abundance and studying the ecology of jaguars (Panthera onca). Ph.D. Dissertation. University of Southampton, Southampton, UK.

Maffei, L., E. Cuellar, and A. Noss. 2004. One thousand jaguars (*Panthera onca*) in Bolivia's Chaco? Camera trapping in the Kaa-Iya National Park. Journal of Zoology **262**:295-304.

Maffei, L., A. J. Noss, S. C. Silver, and M. J. Kelly. 2011. Abundance/Density Case Study: Jaguars in the Americas. Pages 163-190 *in* A. F. O'Connell, J. D. Nichol, and K. U. Karanth, editors. Camera Traps in Animal Ecology: Methods and Analyses. Springer, New York.

Maffei, L., R. Paredes, F. Aguanta, and A. Noss. 2006. Muestreo con trampas cámaras de jaguares y otros mamíferos en la estación Isoso (18° 25’ s, 61° 46’ w) Parque Nacional Kaa Iya del Gran Chaco. 28 de octubre – 24 de diciembre. Wildlife Conservation Society - Fundacion Kaa-Iya, Santa Cruz, Bolivia.

Miller, C. M. 2005. Jaguar density in Gallon Jug Estate, Belize. Wildlife Conservation Society, Gallon Jug, Belize.

Miller, C. M. 2006. Jaguar density in Fireburn, Belize. Wildlife Conservation Society, Belize.

Montaño, R., L. Maffei, and A. Noss. 2007. Segundo muestreo con trampas cámaras de jaguares y otros mamíferos en el Campamento Palmar de las Islas y Ravelo (Diciembre 2006–Marzo 2007). Wildlife Conservation Society, Santa Cruz, Bolivia.

Moreira, J., R. García, R. McNab, G. P. Santizo, M. Mérida, V. Méndez, G. Ruano, M. Córdova, F. Córdova, Y. López, E. Castellanos, R. Lima, and M. Burgos. 2010. Abundancia de jaguares y evaluación de presas asociadas al fototrampeo en las Concesiones Comunitarias del Bloque de Melchor de Mencos, Reserva de la Biosfera Maya, Petén, Guatemala. Wildlife Conservation Society-Programa para Guatemala, Guatemala.

Moreira, J., R. García, R. B. McNab, G. Ruano, G. Ponce, M. Mérida, K. Tut, P. Díaz, E. González, M. Córdova, E. Centeno, C. López, A. Vanegas, Y. Vanegas, F. Córdova, J. Kay, G. Polanco, and M. Barnes. 2005. Abundancia de jaguares y presas asociadas al fototrampeo en el sector oeste del Parque Nacional Mirador - Río Azul, Reserva de Biosfera Maya. Wildlife Conservation Society-Programa para Guatemala, Guatemala.

Moreira, J., R. McNab, R. García, G. Ponce, M. Mérida, V. Méndez, M. Córdova, G. Ruano, K. Tut, H. Tut, F. Córdova, E. Muñoz, E. González, J. Cholom, and A. Xol. 2009. Abundancia y densidad de jaguares en el Parque Nacional Laguna del Tigre-Corredor Biológico Central, Reserva de la Biosfera Maya. Wildlife Conservation Society-Programa para Guatemala, Guatemala.

Moreira, J., R. B. McNab, R. García, V. Méndez, M. Barnes, G. Ponce, A. Vanegas, G. Ical, E. Zepeda, I. García, and M. Córdova. 2008a. Densidad de jaguares dentro de la Concesión Comunitaria de Carmelita y de la Asociación Forestal Integral San Andrés Petén, Guatemala., Wildlife Conservation Society - Jaguar Conservation Program, Guatemala.

Moreira, J., R. B. McNab, R. García, V. Méndez, G. Ponce-Santizo, M. Córdova, S. Tun, T. Caal, and J. Corado. 2008b. Densidad de jaguares en el Biotopo Protegido dos Lagunas, Parque Nacional Mirador Rio Azul, Petén, Guatemala., Wildlife Conservation Society - Jaguar Conservation Program, Guatemala.

Moreira, J., R. B. McNab, D. Thornton, R. García, V. Méndez, A. Vanegas, G. Ical, E. Zepeda, R. Senturión, I. García, J. Cruz, G. Asij, G. Ponce, J. Radachowsky, and M. Córdova. 2007. Abundancia de jaguares en La Gloria-El Lechugal, Zona de Usos Múltiples, Reserva de la Biosfera Maya, Petén, Guatemala. Wildlife Conservation Society-Programa para Guatemala, Guatemala.

Moreno, R. S. R. 2006. Parámetros poblacionales y aspectos ecológicos de los felinos y sus presas en Cana, Parque Nacional Darien, Panamá. M.Sc. Thesis. Universidad Nacional, Heredia, Costa Rica.

Paviolo, A., C. D. de Angelo, Y. E. Di Blanco, and M. S. Di Bitetti. 2008. Jaguar Panthera onca population decline in the Upper Parana Atlantic Forest of Argentina and Brazil. Oryx **42**:554-561.

Payan, E. C. G. 2009. Hunting sustainability, species richness and carnivore conservation in Colombian Amazonia. Ph.D. Dissertation. University College London, London, UK.

Portillo Reyes, H. O. P. and J. Hernández. 2011. Densidad del jaguar (Panthera onca) en honduras: primer estudio con trampas-cámara en la Mosquitia Hondureña. Revista Latinoamericana de Conservación **2**:45-50.

Rojas, R. A. 2006. El jaguar (*Panthera onca*) en el sector San Christobal del Área de Conservación Guanacaste-Costa Rica:densidad, abundancia de presas y depredación de ganado. M.Sc. Thesis. Universidad Nacional, Heredia, Costa Rica.

Romero-Muñoz, A., R. Montaño, R. Peña, T. Dosapey, and R. Paredes. 2006. Muestreo con trampas cámara de jaguares y otros mamíferos en el Campamento Palmar de las Islas (19º 25’ S, 60º 32’ O) 6 de marzo – 6 de junio 2006. Wildlife Conservation Society, Santa Cruz, Bolivia.

Romero-Muñoz, A., R. Paredes, and L. Maffei. 2007. Segundo muestreo con trampas cámaras de jaguares y otros mamíferos en la estación Isoso (18° 25’ s, 61° 46’ w) Parque Nacional Kaa Iya del Gran Chaco.7 de Julio – 8 de Septiembre, 2006. Wildlife Conservation Society - Fundacion Kaa-Iya, Santa Cruz, Bolivia.

Rosas-Rosas, O. C. 2006. Ecological status and conservation of jaguars (*Panthera onca*) in northeastern Sonora, Mexico. Ph.D. Dissertation. New Mexico State University, Las Cruces, New Mexico.

Rumiz, D. I., R. Arispe, A. J. Noss, and K. Rivero. 2003. Censo de jaguares (Panthera onca) y otros mamíferos con trampas-cámara en la estancia San Miguelito, Santa Cruz, Bolivia. Wildlife Conservation Society, Santa Cruz, Bolivia.

Salom-Perez, R., E. Carrillo, J. C. Saenz, and J. M. Mora. 2007. Critical condition of the jaguar Panthera onca population in Corcovado National Park, Costa Rica. Oryx **41**:51-56.

Silveira, L. 2004. Ecologia comparada e conservação da onça-pintada (Panthera onca) e onça-parda (Puma concolor), no Cerrado e Pantanal. Ph.D. Dissertation. Universidade de Brasília, Brasilia, Brazil.

Silveira, L., A. T. A. Jácomo, S. Astete, R. Sollmann, N. M. Tôrres, M. M. Furtado, and J. Marinho-Filho. 2010. Density of the Near Threatened jaguar Panthera onca in the caatinga of north-eastern Brazil. Oryx **44**:104-109.

Silver, S. C., L. E. T. Ostro, L. K. Marsh, L. Maffei, A. M. J. Kelly, R. B. Wallace, H. Gómez, and G. Ayala. 2004. The use of camera traps for estimating jaguar *Panthera onca* abundance and density using capture/recapture analysis. Oryx **38**:148-154.

Soisalo, M. K. and S. M. C. Cavalcanti. 2006. Estimating the density of a jaguar population in the Brazilian Pantanal using camera-traps and capture-recapture sampling in combination with GPS radio-telemetry. Biological Conservation **129**:487-496.

Sollmann, R., M. M. Furtado, B. Gardner, H. Hofer, A. T. A. Jácomo, N. M. Tôrres, and L. Silveira. 2011. Improving density estimates for elusive carnivores: Accounting for sex-specific detection and movements using spatial capture-recapture models for jaguars in central Brazil. Biological Conservation **144**:1017-1024.

Tobler, M. W., S. E. Carrillo-Percastegui, A. H. Zúñiga, and G. Powell. submitted. High jaguar densities and large population sizes in the core habitat of the Southwestern Amazon. Biological Conservation.

Wallace, R. B., H. Gomez, G. Ayala, and F. Espinoza. 2003. Camera trapping for jaguar (*Panthera onca*) in the Tuichi Valley, Bolivia. Journal of Neotropical Mammology **10**:133-139.
