# Supplementary material for: Jaguar Densities across Human-Dominated Landscapes in Colombia: The Contribution of Unprotected Areas to Long Term Conservation
Source: PLoS One. 2016 May 4;11(5):e0153973. doi: 10.1371/journal.pone.0153973 (PMC4856405; doi:10.1371/journal.pone.0153973)
Supplement: S4 Appendix — (DOCX) [file pone.0153973.s004.docx]

**S4 Appendix. Data, Site-I.**

Table A. Individual jaguars recorded, their sex, and corresponding sampling occasions and camera trap stations at Site-I. Individual 2, 3, 5, and 6 were recoded in the area also in 2012.

| **Individuals** | **Sex** | **Sampling Occasions** | **Camera-trap stations** |
| --- | --- | --- | --- |
| 1 | F | 12 | 1 |
| 1 | F | 14 | 4 |
| 1 | F | 18 | 1 |
| 1 | F | 24 | 1 |
| 1 | F | 45 | 1 |
| 1 | F | 46 | 1 |
| 2 | F | 2 | 10 |
| 2 | F | 6 | 8 |
| 2 | F | 6 | 10 |
| 2 | F | 6 | 11 |
| 2 | F | 7 | 11 |
| 2 | F | 9 | 8 |
| 2 | F | 13 | 7 |
| 2 | F | 20 | 6 |
| 2 | F | 22 | 7 |
| 2 | F | 27 | 7 |
| 2 | F | 30 | 7 |
| 2 | F | 34 | 8 |
| 2 | F | 68 | 7 |
| 3 | M | 4 | 2 |
| 3 | M | 11 | 4 |
| 3 | M | 15 | 1 |
| 3 | M | 16 | 2 |
| 3 | M | 20 | 2 |
| 3 | M | 21 | 2 |
| 3 | M | 41 | 2 |
| 3 | M | 43 | 2 |
| 3 | M | 59 | 4 |
| 3 | M | 62 | 4 |
| 4 | F | 14 | 1 |
| 4 | F | 34 | 1 |
| 4 | F | 48 | 2 |
| 4 | F | 54 | 3 |
| 5 | M | 1 | 6 |
| 5 | M | 2 | 7 |
| 5 | M | 6 | 4 |
| 5 | M | 9 | 7 |
| 5 | M | 11 | 6 |
| 5 | M | 17 | 2 |
| 5 | M | 17 | 7 |
| 5 | M | 30 | 2 |
| 5 | M | 30 | 7 |
| 5 | M | 41 | 7 |
| 5 | M | 42 | 5 |
| 5 | M | 42 | 9 |
| 5 | M | 43 | 2 |
| 5 | M | 44 | 2 |
| 5 | M | 55 | 7 |
| 5 | M | 57 | 1 |
| 5 | M | 57 | 2 |
| 5 | M | 59 | 4 |
| 5 | M | 63 | 13 |
| 5 | M | 67 | 2 |
| 5 | M | 67 | 4 |
| 5 | M | 67 | 5 |
| 5 | M | 68 | 5 |
| 5 | M | 71 | 7 |
| 5 | M | 71 | 13 |
| 5 | M | 73 | 1 |
| 6 | F | 30 | 2 |
| 6 | F | 31 | 2 |
| 6 | F | 46 | 13 |
| 6 | F | 68 | 13 |
| 6 | F | 71 | 15 |
| 6 | F | 74 | 14 |
| 7 | F | 20 | 2 |
| 7 | F | 21 | 2 |
| 7 | F | 35 | 2 |
| 7 | F | 38 | 2 |
| 7 | F | 41 | 2 |
| 7 | F | 42 | 2 |
| 7 | F | 43 | 2 |
| 7 | F | 44 | 2 |
| 8 | M | 15 | 2 |
| 8 | M | 18 | 2 |
| 8 | M | 40 | 12 |
| 9 | F | 62 | 7 |
| 9 | F | 63 | 7 |
| 9 | F | 63 | 9 |
| 9 | F | 64 | 7 |
| 9 | F | 65 | 7 |
| 9 | F | 67 | 7 |
| 9 | F | 68 | 5 |
| 9 | F | 68 | 7 |
| 9 | F | 71 | 7 |
| 9 | F | 74 | 1 |
| 9 | F | 74 | 13 |
| 10 | F | 1 | 6 |

Table B. Camera trap stations’ coordinates at Site-I (UTM 18N, WGS 84). Jaguars were recorded at stations 1-15.

| **Camera-trap stations** | **Y-coordinate** | **X-coordinate** |
| --- | --- | --- |
| 1 | 626241 | 821124 |
| 2 | 628096 | 822159 |
| 3 | 630659 | 820465 |
| 4 | 626294 | 822011 |
| 5 | 632114 | 824646 |
| 6 | 633160 | 827230 |
| 7 | 632455 | 825707 |
| 8 | 633764 | 824180 |
| 9 | 632654 | 826481 |
| 10 | 636500 | 825646 |
| 11 | 635687 | 827125 |
| 12 | 626600 | 819757 |
| 13 | 629468 | 822244 |
| 14 | 629391 | 821372 |
| 15 | 630799 | 822423 |
| 16 | 637277 | 831489 |
| 17 | 638328 | 829145 |
| 18 | 639418 | 831981 |
| 19 | 642126 | 827984 |
| 20 | 638064 | 833703 |
| 21 | 640744 | 828754 |
| 22 | 641324 | 832219 |
| 23 | 639381 | 830346 |
| 24 | 634371 | 828064 |
| 25 | 639655 | 827566 |
| 26 | 633057 | 824555 |
| 27 | 634722 | 824427 |
| 28 | 641182 | 826735 |
| 29 | 640781 | 825164 |
| 30 | 636495 | 828982 |
| 31 | 631780 | 823333 |
| 32 | 639270 | 826218 |
| 33 | 637417 | 826610 |
| 34 | 638046 | 824568 |
| 35 | 628116 | 818708 |
| 36 | 624654 | 815380 |
| 37 | 625626 | 816381 |
| 38 | 632892 | 821357 |
| 39 | 632715 | 823179 |
| 40 | 625296 | 818486 |
| 41 | 623355 | 818011 |
| 42 | 624956 | 820910 |
| 43 | 623151 | 820697 |
| 44 | 627314 | 817668 |
| 45 | 632621 | 819831 |
| 46 | 631872 | 821419 |
| 47 | 629576 | 820328 |
