# Supplementary material for: Jaguar Densities across Human-Dominated Landscapes in Colombia: The Contribution of Unprotected Areas to Long Term Conservation
Source: PLoS One. 2016 May 4;11(5):e0153973. doi: 10.1371/journal.pone.0153973 (PMC4856405; doi:10.1371/journal.pone.0153973)
Supplement: S5 Appendix — (DOCX) [file pone.0153973.s005.docx]

**S5 Appendix. Data, Site-II.**

Table A. Individual jaguars recorded, their sex, and corresponding sampling occasions and camera trap stations at Site-II.

| **Individuals** | **Sex** | **Sampling Occasions** | **Camera-trap stations** |
| --- | --- | --- | --- |
| 1 | M | 2 | 1 |
| 1 | M | 3 | 1 |
| 1 | M | 6 | 1 |
| 1 | M | 7 | 1 |
| 1 | M | 13 | 1 |
| 1 | M | 17 | 1 |
| 1 | M | 18 | 1 |
| 1 | M | 24 | 1 |
| 1 | M | 35 | 1 |
| 1 | M | 49 | 1 |
| 1 | M | 9 | 2 |
| 1 | M | 12 | 4 |
| 1 | M | 13 | 4 |
| 1 | M | 17 | 4 |
| 1 | M | 18 | 4 |
| 1 | M | 21 | 4 |
| 1 | M | 23 | 4 |
| 1 | M | 26 | 4 |
| 1 | M | 27 | 4 |
| 1 | M | 29 | 4 |
| 1 | M | 34 | 4 |
| 1 | M | 35 | 4 |
| 1 | M | 40 | 4 |
| 1 | M | 47 | 4 |
| 1 | M | 49 | 4 |
| 1 | M | 29 | 7 |
| 1 | M | 37 | 8 |
| 1 | M | 41 | 11 |
| 2 | M | 1 | 1 |
| 2 | M | 2 | 1 |
| 2 | M | 4 | 1 |
| 2 | M | 4 | 2 |
| 2 | M | 7 | 2 |
| 2 | M | 8 | 2 |
| 2 | M | 14 | 2 |
| 2 | M | 21 | 2 |
| 2 | M | 5 | 3 |
| 2 | M | 56 | 3 |
| 2 | M | 56 | 4 |
| 2 | M | 59 | 4 |
| 2 | M | 60 | 4 |
| 2 | M | 62 | 4 |
| 2 | M | 66 | 4 |
| 2 | M | 67 | 4 |
| 2 | M | 68 | 4 |
| 3 | M | 1 | 1 |
| 3 | M | 8 | 1 |
| 3 | M | 9 | 1 |
| 3 | M | 20 | 1 |
| 3 | M | 22 | 1 |
| 3 | M | 22 | 4 |
| 3 | M | 33 | 4 |
| 3 | M | 41 | 4 |
| 3 | M | 44 | 4 |
| 3 | M | 63 | 4 |
| 3 | M | 19 | 5 |
| 3 | M | 40 | 10 |
| 4 | F | 18 | 1 |
| 4 | F | 16 | 6 |
| 4 | F | 67 | 14 |
| 5 | M | 15 | 1 |
| 5 | F | 8 | 2 |
| 5 | F | 54 | 2 |
| 5 | F | 59 | 2 |
| 5 | F | 37 | 9 |
| 6 | NA | 66 | 13 |

Table B. Camera trap stations’ coordinates at Site-II (UTM 19N, WGS 84). Jaguars were recorded at stations 1-11, 13, and 14.

| **Camera-trap stations** | **Y-coordinate** | **X-coordinate** |
| --- | --- | --- |
| 1 | 236194 | 668956 |
| 2 | 232846 | 667642 |
| 3 | 236361 | 669743 |
| 4 | 235790 | 668607 |
| 5 | 240073 | 672335 |
| 6 | 234122 | 669182 |
| 7 | 237410 | 670383 |
| 8 | 233023 | 666941 |
| 9 | 231635 | 663250 |
| 10 | 238831 | 672113 |
| 11 | 230703 | 666818 |
| 12 | 240765 | 670784 |
| 13 | 241997 | 667420 |
| 14 | 237822 | 668111 |
| 15 | 235302 | 664165 |
| 16 | 237685 | 663167 |
| 17 | 240942 | 663665 |
| 18 | 233866 | 662949 |
| 19 | 241883 | 672730 |
| 20 | 230866 | 662205 |
| 21 | 232502 | 665234 |
| 22 | 239071 | 664530 |
| 23 | 234498 | 666085 |
| 24 | 230789 | 665064 |
| 25 | 238873 | 670242 |
| 26 | 226886 | 659150 |
| 27 | 243381 | 671188 |
| 28 | 244749 | 669700 |
| 29 | 234526 | 660551 |
| 30 | 244269 | 666728 |
| 31 | 245695 | 668156 |
| 32 | 237237 | 665063 |
| 33 | 243811 | 668644 |
| 34 | 229036 | 658873 |
| 35 | 240705 | 666064 |
| 36 | 240534 | 666615 |
| 37 | 242914 | 665106 |
| 38 | 244803 | 664434 |
| 39 | 239964 | 668786 |
| 40 | 230498 | 660287 |
| 41 | 226264 | 662263 |
| 42 | 232092 | 661664 |
| 43 | 232240 | 659667 |
| 44 | 242305 | 669509 |
| 45 | 229550 | 663021 |
| 46 | 227125 | 661331 |
| 47 | 238818 | 666438 |
| 48 | 236348 | 666988 |
| 49 | 227921 | 663569 |
| 50 | 228381 | 660770 |
| 51 | 225060 | 660316 |
| 52 | 235854 | 661996 |
